# Impact of psychotropic drugs on cognitive impairment in type I bipolar disorder

**DOI:** 10.1192/j.eurpsy.2026.11191

**Published:** 2026-07-31

**Authors:** N. Smaoui, H. Sghaier, L. Triki, R. Feki, I. Gassara, S. Omri, M. Maalej, M. Maalej, N. Charfi, L. Zouari

**Affiliations:** 1 Psychiatry “C”, Hedi Chaker University Hospital; 2 Department of Functional Explorations, Habib Bourguiba University Hospital, Sfax, Tunisia

## Abstract

**Introduction:**

Cognitive impairment is a frequent feature in bipolar disorder type 1 (BD-1). While the etiology of cognitive dysfunction in BD-1 is multifactorial, psychotropic medications, including mood stabilizers and antipsychotics, may contribute to cognitive changes. Understanding the relationship between therapeutic factors and cognitive performance is crucial for optimizing treatment strategies and preserving cognitive capacities in bipolar disorder.

**Objectives:**

The aim of this study was to assess the relationship between cognitive impairment and therapeutic factors, particularly the use of psychotropic drugs, in patients with bipolar disorder type 1 (BD-1).

**Methods:**

This was a cross-sectional, case-control study. It was carried out at the “C” psychiatry outpatient unit at CHU Hedi Chaker, Sfax, Tunisia. Fifteen TB-1 patients and fifteen healthy controls were included. All participants were assessed by the Screen For Cognitive Impairment in Psychiatry (SCIP) scale for evaluation of cognitive functions.

**Results:**

All cases were receiving at least one mood stabilizer at the time of the study, of which the most prescribed molecule was sodium valproate (n=8; 53.4%). At the time of the study, all cases were receiving at least one antipsychotic (AP). Eight were receiving a first-generation AP (53.3%) and 10 were receiving a second-generation AP (66.7%), the two prescribed being risperidone (n=7; 46.7%) and olanzapine (n=3; 20%). The mean SCIP score was significantly lower in patients on sodium valproate (34.3 ± 9.9) than in those not receiving this molecule (49.7 ± 9.1). The analytical study found that the SCIP total score and the subscores of working memory (WMT) and long-term verbal memory (LTV-D) were significantly lower in cases treated with sodium valproate than in those who did not receive it (p=0.008; p= 0.021; p=0.013).

No significant correlation was observed between SCIP scores and other psychotropic drug classes

**Conclusions:**

Patients with TB-1 taking sodium valproate showed specific cognitive impairments (reduced WMT and VLT-D subscores), in contrast to other psychotropic drugs. These results underline the need to monitor cognitive function in patients on this type of treatment, and call for longitudinal studies to further investigate its impact.

**Disclosure of Interest:**

None Declared

